# Advances in In Vitro Diagnostics for Cholangiocarcinoma: From Biomarker Discovery to Artificial Intelligence

**DOI:** 10.3390/ijms27093779

**Published:** 2026-04-23

**Authors:** Chengrui Mo, Xinping Hu, Zhu Yuan, Tiancai Liu

**Affiliations:** Key Laboratory of Antibody Engineering of Guangdong Higher Education Institutes, School of Laboratory Medicine and Biotechnology, Southern Medical University, Guangzhou 510515, China; 13711999807@163.com (C.M.); 15303064758@163.com (X.H.); yuanxiaoranz@icloud.com (Z.Y.)

**Keywords:** cholangiocarcinoma, liquid biopsy, circulating tumor cells, circulating tumor DNA, extracellular vesicles, artificial intelligence

## Abstract

Cholangiocarcinoma (CCA) is a highly aggressive malignancy that is difficult to diagnose early and carries a poor prognosis. Conventional serological diagnostics are limited by inadequate sensitivity and the risks of invasive procedures, resulting in most patients being diagnosed at an advanced stage. In recent years, liquid biopsy has emerged as a novel approach for non-invasive and dynamic molecular tumor monitoring by detecting biomarkers such as circulating tumor cells (CTCs), extracellular vesicles (EVs), circulating tumor DNA (ctDNA) and clusterin (CLU). Furthermore, artificial intelligence (AI) has demonstrated strong potential in aiding diagnosis through medical image analysis, pathological pattern recognition, and multi-omics data integration, significantly improving the precision of early detection, risk stratification, and treatment response monitoring in CCA. This review systematically summarizes recent advances in liquid biopsy and AI for CCA diagnosis, discusses their clinical potential and current challenges, and offers perspectives on how their integration can propel the field toward earlier and more precise management of the disease.

## 1. Introduction

**Figure 1:**
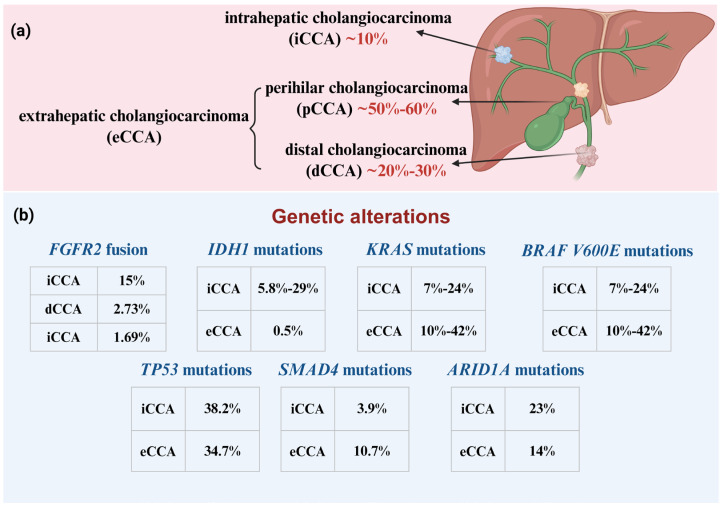
Anatomical distribution and molecular landscape of cholangiocarcinoma subtypes. (**a**) Schematic representation of the anatomical classification of CCA and the approximate proportion of each subtype: iCCA ~10%; pCCA ~50–60% and dCCA ~40% [3,4]; (**b**) Frequency of key driver gene alterations across CCA subtypes (%). *FGFR2* fusions, *IDH1* mutations, and *ARID1A* alterations are enriched in iCCA, while *KRAS* and *SMAD4* mutations are more common in eCCA. *TP53* and *BRAF V600E* mutations occur at similar frequencies in both subtypes. *BRAF V600E* mutations occur at low frequency across all subtypes [5,6,7,8,9]. Created in BioRender. Mo, C. (2026) https://BioRender.com/uxr842c.

Cholangiocarcinoma is a highly malignant gastrointestinal tumor originating from the biliary epithelium. Although it accounts for only 3% of gastrointestinal cancers, its global incidence is increasing rapidly at a rate of 2% to 3% per year. Due to the challenges of early diagnosis, frequent misdiagnosis, and limited treatment options, CCA has become one of the malignant tumors with the poorest prognosis [1]. Based on anatomical location, histological characteristics, and growth pattern, cholangiocarcinoma is classified into subtypes: intrahepatic cholangiocarcinoma (iCCA), perihilar cholangiocarcinoma (pCCA), and distal cholangiocarcinoma (dCCA). Mixed hepatocellular cholangiocarcinoma is recognized as a distinct subtype of primary liver cancer [2]. Extrahepatic cholangiocarcinoma (eCCA) encompasses both pCCA and dCCA subtypes [3]. The molecular landscape of CCA varies markedly by anatomical location, with distinct driver alterations enriched in intrahepatic versus extrahepatic subtypes, as summarized in Figure 1. These subtype-specific genetic events not only refine molecular classification but also provide critical targets for liquid biopsy and precision oncology.

Currently, the clinical diagnosis of cholangiocarcinoma still relies on the combined use of imaging studies, serum tumor marker testing, and histopathological examination. For patients with suspected biliary strictures, abdominal ultrasound (US) is the preferred initial screening method due to its non-invasive nature; however, because of the complex anatomical structure and susceptibility to interference from intestinal gas, obtaining a complete view of the extrahepatic bile ducts is often challenging [10]. Computed tomography (CT) is the primary tool for tumor staging and assessment of resectability, but it has limitations in depicting the complex anatomy of the hilar biliary tract [11]. Magnetic resonance imaging (MRI) offers excellent soft tissue resolution but is contraindicated in patients with certain metallic implants [12,13]. Serum CA19-9 and CEA are commonly used as adjunctive markers [14,15]. The diagnostic performance and specific limitations of the aforementioned traditional methods are summarized in Table 1. Pathological biopsy, as the diagnostic gold standard, can definitively establish the nature and subtype of the lesion. However, it is an invasive procedure that may cause bleeding, bile leakage, or even needle-track seeding. Moreover, it is associated with a high false-negative rate in extrahepatic CCA [16,17].

Traditional diagnostic methods have limited sensitivity and specificity in the early detection of cholangiocarcinoma, which is the primary cause of misdiagnosis, delayed diagnosis, and missed opportunities for optimal treatment. This review, therefore, systematically explores the application prospects of liquid biopsy and AI in the management of CCA. Liquid biopsy offers a non-invasive approach for dynamic monitoring by detecting biomarkers such as CTCs, EVs, ctDNA, and other circulating analytes, thereby facilitating early diagnosis, prognostic evaluation, and recurrence surveillance. Simultaneously, AI technology has demonstrated substantial value in medical image analysis, pathological pattern recognition, and the big-data analysis of disease biomarkers. It enables the precise interpretation and pattern recognition of the multidimensional data derived from liquid biopsy, collectively forming a synergistic “data acquisition-intelligent analysis” closed loop. This review aims to summarize the current progress in applying these technologies to CCA serological testing, analyze the challenges hindering their clinical translation, and outline future directions, with the ultimate goal of promoting optimized strategies for early screening and precision therapy in CCA.

## 2. Methods

We conducted a comprehensive literature search in PubMed, Web of Science, and Scopus for articles published up to April 2026. A total of 96 relevant articles were retrieved and systematically reviewed. The search strategy combined terms related to cholangiocarcinoma (“cholangiocarcinoma” OR “bile duct cancer” or “CCA”) with terms related to liquid biopsy: (“liquid biopsy”), (“circulating tumor cells” OR “CTCs”), (“extracellular vesicles” OR “exosomes”), (“circulating tumor DNA” OR “ctDNA”), (“clusterin” OR “CLU”) with terms related to artificial intelligence (“artificial intelligence” or “machine learning” or “deep learning” or “AI”). Additionally, other literature was identified through manual screening of reference lists from relevant reviews and original studies. Articles were included in this study if they reported original data on the diagnostic performance, technological advances, or clinical applications of liquid biopsy or artificial intelligence in cholangiocarcinoma (CCA). Editorials and non-English publications were excluded. Data extraction focused on key findings, biomarker types, analytical platforms, and reported diagnostic accuracy metrics.

## 3. Liquid Biopsy: A Breakthrough in Tumor Biomarkers for Early Diagnosis

Liquid biopsy enables the non-invasive detection of tumor markers, including CTCs, EVs, and ctDNA, from bodily fluids such as blood and saliva. This approach offers the advantages of simple, repeatable sampling and can comprehensively profile the molecular landscape of tumors. It is applicable for early cancer screening, treatment response assessment, drug resistance monitoring, and minimal residual disease detection, thereby informing personalized therapy and shifting the cancer treatment paradigm toward earlier stages [23].

### 3.1. Circulating Tumor Cell, CTCs

CTCs are cancer cells that detach from primary or metastatic tumors and enter the bloodstream. They serve as important biomarkers for cancer screening, prognosis assessment, and treatment monitoring.

The presence of CTCs has been demonstrated to correlate with poor prognosis in patients with breast cancer, prostate cancer, and colorectal cancer [24,25,26]. Based on this, Yang Ju Dong’s research team hypothesized that CTCs are similarly associated with poor survival in CCA patients. Using the CellSearch system, the study successfully detected epithelial cell adhesion molecule (EpCAM)-positive CTCs in the peripheral blood of CCA patients, confirming the feasibility of capturing and identifying tumor cells of cholangiocarcinoma origin from blood. The study found that CTC positivity (defined as ≥ 2 cells/7.5 mL blood) was significantly associated with more aggressive tumor phenotypes, including larger tumor size, multinodular growth, bilateral intrahepatic invasion, lymph node metastasis, and distant metastasis. This indicates that CTC levels effectively reflect tumor burden and invasive metastatic potential in vivo. Furthermore, CTCs serve as an independent prognostic predictor for overall survival in CCA patients. In multivariate Cox analysis, CTC presence remained an independent risk factor for shorter survival even after adjusting for traditional prognostic factors like distant metastasis, CA19-9 levels, and age, demonstrating CTCs’ independent predictive capability in prognosis assessment. Moreover, this prognostic predictive value remains significant in key subgroups such as metastatic patients and those with hilar/distal CCA, suggesting the potential clinical utility of CTC detection across diverse settings. In summary, CTCs in cholangiocarcinoma can be reliably detected, their quantitative levels reflect tumor malignancy, and they provide prognostic information independent of traditional markers. Collectively, these findings constitute compelling evidence for incorporating CTCs into the liquid biopsy system for CCA [27].

The current method for detecting CTCs employs Immunomagnetic Separation to enrich circulating tumor cells. This involves collecting peripheral venous blood from patients, diluting it with PBS buffer, and removing impurities through gradient centrifugation. Antibody-conjugated magnetic beads are used to target and capture CTCs, while unbound components are removed via magnetic separation. Enriched cells are resuspended in buffer and undergo automated fluorescent staining and cell imaging using the Cell Tracks Analyzer system. Finally, the results are interpreted by a laboratory physician. The Cell Tracks Analyzer system has undergone preliminary clinical validation, is supported by prospective studies, demonstrates prognostic value across multiple cancer types, and serves as a foundational platform for downstream analysis. This system represents one of the emerging technologies in modern medicine [28].

The low abundance of CTCs in peripheral blood has long posed a significant technical challenge for their accurate enumeration. Consequently, the detection of CTCs requires continuous development of new methods to improve sensitivity and specificity. Recently, Wang’s research group proposed a novel microdroplet-based digital method for absolute quantification of CTCs. This technique employs a microfluidic chip to partition whole blood into tens of thousands of nanoliter reaction droplets, isolating individual CTCs within each droplet. By applying the Poisson distribution to calculate the positive proportion within droplets, the method enables direct single-cell-level CTC counting. Its sensitivity achieves absolute quantification down to 5 CTCs/5 mL whole blood, significantly outperforming current enrichment-dependent platforms. This technology integrates multiplex gene amplification and fluorescence analysis within microdroplets on a single platform, enabling simultaneous detection of CTC counts and genotyping in one assay [29]. This holds promise for precise diagnosis and prognosis monitoring in cancers such as CCA, where early detection is notoriously difficult. Compared with conventional detection methods, microdroplet digital technology provides core advantages of high sensitivity, absolute quantification, high specificity, and strong interference resistance.

Compared to traditional tissue biopsies, CTCs detection via peripheral blood sampling offers the advantages of simplicity and minimal invasiveness, making it particularly suitable for CCA patients where needle aspiration is challenging. Its high sensitivity aids in the early detection of occult metastases. CTCs comprehensively reflect the molecular characteristics of different metastatic sites, overcoming tumor spatial heterogeneity. By dynamically monitoring their quantity and genetic mutations, it tracks clonal evolution and evaluates treatment efficacy, compensating for temporal heterogeneity [30,31,32]. This detection holds significant value in early warning, precision treatment guidance, and prognosis assessment. Although the combined use of CTCs detection with imaging and serum biomarkers enhances the value of tumor burden assessment and staging prognosis, its standalone application for early screening and diagnosis still requires AI-assisted validation in large-scale studies and refinement of standards. It should be noted, however, that the current evidence for CTCs in CCA derives primarily from cohorts that include both intrahepatic and extrahepatic subtypes, and whether CTC enumeration or phenotypic profiles differ systematically between iCCA and eCCA remains an open question that warrants further subtype-stratified investigation [27].

### 3.2. Extracellular Vesicles, EVs

EVs are actively secreted by cells as lipid bilayer membrane structures ranging from 10 to 1000 nm in diameter. They contain diverse biomolecules, including proteins, lipids, DNA, RNA (such as mRNA, miRNA, and lncRNA), and even viruses or prions, serving as crucial mediators of intercellular communication [33]. EVs are broadly categorized into two types: ectosomes and exosomes [34]. Cholangiocarcinoma cells exhibit markedly enhanced secretion of EVs, releasing quantities exceeding tenfold that of normal cholangiocarcinoma epithelial cells. These tumor-derived EVs carry diverse oncogenic molecular payloads (e.g., oncogene proteins, pro-metastatic miRNAs), which, upon delivery to recipient cells, participate in reprogramming the tumor microenvironment, thereby promoting the invasion and metastasis of cholangiocarcinoma [35]. Furthermore, bile—as a non-circulatory fluid directly contacting biliary tract tumors—can specifically enrich tumor-derived EVs in the upstream region of strictures. Unlike the oncogenic properties exhibited by EVs in malignant strictures, those in benign strictures (e.g., caused by chronic inflammation or stones) primarily reflect local inflammatory states. These two types of EVs not only differ significantly in concentration but also exhibit fundamental functional distinctions. Li et al. demonstrated that extracellular vesicles isolated from human bile are selectively enriched with a distinct subset of miRNAs compared to the supernatant bile fraction. Using a panel of five EV-derived miRNAs, they were able to differentiate CCA from benign biliary conditions—including primary sclerosing cholangitis, benign biliary stricture, and choledocholithiasis—with a sensitivity of 67% and a specificity of 96% [36]. Consequently, recent studies have explored using EVs as a reliable diagnostic marker for CCA. Valeria Severino’s research team demonstrated that measuring bile-derived EVs can accurately distinguish malignant from benign biliary strictures [37]. Given that bile is in direct contact with the biliary epithelium, bile-derived EVs offer unique diagnostic advantages for eCCA and other biliary tract cancers, with studies demonstrating superior diagnostic performance of bile EVs over serum EVs. For iCCA embedded within the liver parenchyma, the diagnostic utility of bile EVs remains less established, and serum-derived EVs have shown promise as an alternative liquid biopsy source for this subtype [36,38,39]. The following Table 2 summarizes findings from their experiments:

Experimental studies suggest that bile-derived EVs show significant potential for the early diagnosis, prognostic evaluation, and targeted treatment of CCA. This parallels the growing recognition of specific proteins carried by serum EVs as valuable diagnostic biomarkers for CCA. As shown in Table 3, these proteins have been demonstrated to play a definitive role in the differential diagnosis and risk prediction of CCA. Diagnostic models constructed based on EV proteins and their combinations hold promise for providing more precise directions for the clinical detection of CCA.

Exosomes, with diameters ranging from approximately 40 to 160 nm, originate from intracellular multivesicular bodies. Following fusion with the cell membrane, they are released into the extracellular matrix [34]. The pivotal role of non-coding RNAs carried by exosomes in the development, metastasis, and immune regulation of cholangiocarcinoma represents a current research focus [41]. Table 4 summarizes three representative types of exosomal non-coding RNAs derived from various bodily fluids such as serum, tissue, or bile. These provide key molecular evidence for establishing a non-invasive, precise diagnostic and prognostic evaluation system for cholangiocarcinoma.

Owing to the characteristics of exosomal circRNA—including circularization, stability, high conservation, and characteristic enrichment in extracellular vesicles [45]—it is garnering increasing attention as a liquid biopsy biomarker for cholangiocarcinoma. Based on liquid biopsy samples combining serum and bile, West China Hospital of Sichuan University developed a diagnostic and dynamic monitoring system for cholangiocarcinoma centered on exosomal circRNA. They discovered that tumor microenvironment-derived exosomes (functional molecules such as circ-CCAC1 and circ-PTPN22) participate in tumor invasion and angiogenesis by mediating intercellular communication. The research team employed a multi-modal approach integrating differential ultracentrifugation, transmission electron microscopy, nanoparticle tracking analysis, and Western blot techniques for the separation and characterization of serum- and bile-derived exosomes, which is the most commonly used method in clinical practice for detecting bile EVs. Furthermore, through a combination of gene chip high-throughput screening and divergent primer quantitative PCR, the team has, for the first time, jointly identified a combination of circled RNA biomarkers (hsa-circ-0000367, hsa-circ-0021647, and hsa-circ-0000288) from both bile and serum. Their circularization characteristics were validated via RNase R digestion followed by Sanger sequencing. The primary innovation of this assay lies in combining bile’s localization specificity for biliary tract lesions with serum’s ease of sampling, forming complementary detection pathways. This offers an enhanced alternative to traditional tumor markers for improving early diagnosis efficacy and follow-up monitoring, which is one of the emerging technologies in modern medicine [46].

EVs harboring bile-specific or serum-specific proteins, such as bile-derived microRNAs or serum-specific proteins, possess the potential to serve as novel markers for cholangiocarcinoma, thereby enabling earlier diagnosis and differential diagnosis of this malignancy. The clinical application of EVs in cholangiocarcinoma diagnosis holds considerable value. Biliary EVs exhibit higher concentrations than CA19-9 [37] and demonstrate high diagnostic utility for malignant biliary strictures.

Although EVs hold promise for playing a significant role in the diagnosis and treatment of cholangiocarcinoma, several challenges remain before their translation into clinical applications. Presently, the mechanisms governing the biogenesis and secretion of EVs, as well as the precise mechanisms underlying EV-mediated intercellular communication, remain unclear. Concurrently, existing methods for EV extraction, purification, and cryopreservation lack standardization, readily leading to inconsistent detection values. This severely constrains the further advancement of their clinical translational application [47].

### 3.3. Circulating Tumor DNA, ctDNA

CtDNA refers to free DNA fragments (circulating free DNA, cfDNA) released into the peripheral bloodstream by tumor cells through processes such as apoptosis or necrosis. It primarily originates from the primary tumor, CTCs, micrometastases, or overt metastatic lesions. As direct derivatives of tumor cell genetic material, ctDNA fragments carry genetic variation information, including mutations specific to their tumor cell of origin [48].

Dr James Yu and his research team conducted dynamic ctDNA analysis on 195 plasma samples from 56 cholangiocarcinoma patients (median follow-up 12.8 months), demonstrating that tumor-specific ctDNA monitoring can be utilized for prognostic prediction and recurrence surveillance. Results indicated that ctDNA positivity during the postoperative molecular residual disease (MRD) window and monitoring period significantly predicted shorter recurrence-free survival, suggesting markedly enhanced predictive capability compared to the traditional CA19-9 biomarker. Furthermore, ctDNA demonstrated highly accurate monitoring of cholangiocarcinoma recurrence, detecting 16 recurrent cases (93.8%) in the study cohort. Compared to imaging-confirmed recurrence, changes in ctDNA levels predicted recurrence on average 3.7 months earlier; Furthermore, ctDNA status can explain false-positive findings detected by imaging. Among 37 patients initially flagged as positive by imaging but ultimately confirmed as non-recurrent, ctDNA testing was consistently negative, and subsequent diagnosis confirmed no recurrence [49].

Common techniques employed in ctDNA research include droplet digital PCR (ddPCR) and next-generation sequencing (NGS), each offering distinct advantages in detection principles, sensitivity, specificity, and throughput.

NGS offers an excellent approach for precision cancer therapy through ctDNA detection. The advantages of NGS lie in its ability to perform multi-gene testing on minimally invasive tissue samples, overcoming the limitations of tissue biopsy heterogeneity, and enabling real-time dynamic monitoring to simultaneously detect treatment responses and the dynamic changes within tumor clonal populations. For instance, “DNA + RNA”-based NGS technology is the preferred approach for detecting *FGFR2* fusions in intrahepatic cholangiocarcinoma. It precisely identifies *FGFR2* fusions and diverse fusion partners, even recognizing complex *FGFR2* fusion patterns, with high concordance (κ value = 0.867) to the gold standard FISH assay and superior detection performance. Simultaneously, this technology enables concurrent detection of *IDH2*, *BRAF*, and other characteristic driver gene mutations in cholangiocarcinoma, enabling comprehensive identification of potential therapeutic targets for patients. Furthermore, NGS-based liquid biopsy offers a minimally invasive *FGFR2* fusion detection method for advanced cholangiocarcinoma patients with insufficient tissue samples, expanding its clinical applications and providing critical, comprehensive molecular diagnostic evidence for targeted therapy in cholangiocarcinoma [50]. However, NGS utilization faces certain limitations. For instance, ctDNA levels are extremely low in early-stage patients, with clinical sensitivity falling below 50%, thereby restricting its widespread application in early diagnosis [51]. It is anticipated that future refinements in standardized NGS testing protocols, coupled with the establishment of clear clinical intervention thresholds for ultra-low-frequency variants, will broaden the applicability of NGS. This expansion will extend its use beyond advanced-stage diagnosis and clinical monitoring to encompass early-stage screening [52].

Given that *FGFR2* fusions and *IDH1/2* mutations are predominantly enriched in iCCA, the clinical utility of ctDNA-based genotyping for these alterations is greatest in the intrahepatic subtype [5,6]. Conversely, for extrahepatic CCA, where *KRAS* and *TP53* mutations are more prevalent [6,7,9], ctDNA assays targeting these genes may offer broader diagnostic and monitoring value, although the overall landscape of ctDNA applications in eCCA remains less extensively characterized.

Currently, ddPCR has demonstrated high sensitivity and quantitative accuracy in the detection of ctDNA in cholangiocarcinoma. Regarding the *IDH1*/*2* mutations present in approximately 30% of cholangiocarcinoma patients, in a study by Morten Lapin et al. involving 31 patients with advanced cholangiocarcinoma and known *IDH* mutations in their tumor tissue, the sensitivity of ddPCR for detecting *IDH* mutations in plasma ctDNA reached 84% (95% CI: 66–95%), which is highly comparable to the detection rate of NGS (83%). The variant allele frequencies (VAF) measured by ddPCR and NGS also showed a significant correlation (correlation coefficient 0.8, *p* < 0.001). This technology is suitable for the dynamic monitoring of targeted mutations; serial blood sampling analysis showed that, compared with patients whose *IDH*-mutated ctDNA levels remained unchanged or increased during treatment, patients with decreased IDH-mutated ctDNA levels exhibited a trend toward prolonged median survival. Additionally, analysis of paired baseline and disease progression samples indicated that the VAF of *IDH*-mutated ctDNA was significantly elevated at the time of progression, suggesting that ddPCR can reflect changes in tumor burden and clonal evolution prior to radiological progression. Although limited to predefined targets, ddPCR remains highly promising for precision oncology in IDH-mutant cholangiocarcinoma given its tissue-validated concordance and capacity for longitudinal monitoring [53].

ddPCR and NGS each have distinct strengths in ctDNA detection: the former excels at tracking known mutations over time, while the latter excels at analysis of unknown pan-mutation profiles. However, the clinical translation of this technology still faces key challenges in standardization and defining indications, requiring further validation through large-scale studies and AI-assisted research. However, positive results detected by liquid biopsy, such as postoperative ctDNA positivity, currently lack standardized clinical response pathways, making it difficult to directly guide the escalation or adjustment of cholangiocarcinoma treatment regimens [54]. By integrating multi-omics data with clinical records, AI models hold promise for developing personalized risk stratification and intervention recommendation frameworks for liquid biopsy positives in the future, thereby bridging the gap between test results and clinical decision-making.

### 3.4. Clusterin, CLU

Bile-based liquid biopsy offers a new approach to the early diagnosis of CCA. Through a multicenter bile proteomics analysis, Gao et al. found that clusterin (CLU) is significantly overexpressed in the bile of CCA patients and validated the diagnostic value of bile CLU in 376 ex vivo samples: AUC = 0.852, sensitivity 73.6%, specificity 90.1%, establishing CLU as a key bile biomarker [55]. Notably, the same research team extended and expanded upon in vitro diagnostic technologies to develop a surface plasmon resonance-based fiber-optic sensor. Although this technology is primarily designed for real-time detection during endoscopy, its detection mechanism is fundamentally based on the antigen–antibody binding reaction of CLU in bile samples, aligning with the principles of in vitro immunoassays [56].

Although CLU shows great potential as an in vitro diagnostic marker for CCA, the following limitations currently exist: First, bile samples must be obtained via endoscopic retrograde cholangiopancreatography (ERCP), an invasive procedure, which limits its application as a tool for large-scale screening [57]. The diagnostic value of CLU in more readily obtainable bodily fluids, such as serum and urine, remains unclear [58]. If detection can be achieved using non-invasive samples, it would greatly expand its application scenarios; Second, the validation cohorts for existing diagnostic models and rapid testing platforms primarily come from a single region, and there is a lack of multicenter, multiethnic external validation [55]. Furthermore, standardized testing procedures for CLU have not yet been established, and the comparability of results across different platforms requires further research.

### 3.5. Summary of Liquid Biopsy in the Diagnosis of CCA

Table 5 provides a concise summary of the liquid biopsy techniques reviewed in this chapter, highlighting their complementary roles in CCA diagnosis.

In the clinical practice of CCA, the widespread adoption of liquid biopsy remains hampered by uncertainties regarding the test’s performance: most existing evidence comes from small-sample retrospective analyses, lacking support from large-scale prospective cohort studies, thereby hindering the implementation of clinical standards [59]. Differences in operational protocols across laboratories regarding sample collection, processing, and analysis further undermine the reproducibility of results, diminishing the value of a test report when compared across different platforms [60].

Specifically regarding the early diagnosis of CCA, the sensitivity of liquid biopsy remains under active investigation, and even under ideal conditions, it cannot fully replace tissue biopsy [61]. Currently, liquid biopsy shows some promise in the diagnosis of CCA; however, data on its use are still being accumulated. As a result, it should be used only as an adjunct to traditional biomarkers and pathological evaluation, rather than as the sole basis for clinical decision-making [62,63]. Beyond these technical challenges, more immediate obstacles include clinicians’ insufficient recognition of the test’s value, low trust in liquid biopsy among patients and the public, limited health insurance coverage, and high testing costs. In some regions, structural disparities within the healthcare system have led to uneven accessibility. The interplay of these factors has prevented a technology capable of accelerating precision diagnosis and treatment from gaining a firm foothold in broader clinical settings [64,65].

To accelerate the clinical translation of liquid biopsy for CCA, rather than persisting with blood as a substitute for tissue, it is better to first clarify the most suitable clinical scenarios for each technology: early diagnosis should focus on EVs derived from bile to compensate for the low abundance of tumor-derived material in plasma, while postoperative recurrence monitoring should focus on dynamic changes in ctDNA. Building on this foundation, establishing a routine closed-loop system for report interpretation involving clinicians, laboratory personnel, and bioinformaticians—together with a two-step approach that uses pan-cancer screening followed by tumor-specific confirmation to spread costs—and further supplemented by prospective, standardized validation tailored to the context of jaundice, may help this test more quickly overcome the challenges of inconsistent performance and confusing interpretation, thereby truly integrating it into the precision diagnosis and treatment workflow for CCA.

## 4. The Application of AI-Assisted Technology in the Diagnosis and Treatment of CCA

### 4.1. The Potential for Employing AI in Clinical Settings to Assist with the Diagnosis of Cholangiocarcinoma

Liquid biopsy technology provides rich biological information, such as CTCs, ctDNA, EVs, and CLUs, for the clinical diagnosis of cholangiocarcinoma through blood and other samples. Yet, identifying clinically meaningful patterns within vast datasets and making accurate clinical diagnoses remains a major challenge. The rapid advancement of AI today offers highly efficient tools for processing these clinical data. Artificial Intelligence (AI), Machine Learning (ML), and Deep Learning (DL) form a progressive relationship [66]. Leveraging its powerful data processing and pattern recognition capabilities, deep learning is propelling the medical field toward a new era of multidimensional, dynamic intelligent diagnosis and treatment. It integrates multi-source information, including liquid biopsy data, to assist in early tumor diagnosis, treatment efficacy assessment, and comprehensive management throughout the disease course [67]. The following sections will detail the practical application value of AI in clinical scenarios such as liquid biopsy, pathological examination, and imaging diagnosis for cholangiocarcinoma.

### 4.2. AI-Assisted Analysis of Liquid Biopsy Data for CCA

#### 4.2.1. An Intelligent Diagnostic Model for CCA Based on Liquid Biopsy

Building on validation at the organizational level, the direct application of artificial intelligence is evident in the intelligent analysis of liquid biopsy data. Based on the aforementioned CLU biomarkers, Gao et al. further developed a CCA diagnostic model using DL that incorporates seven indicators. The model’s core comprises a diagnostic combination of seven indicators, specifically including CLU in bile alongside six conventional serum markers: CA19-9, indirect bilirubin (IBIL), gamma-glutamyl transferase (GGT), low-density lipoprotein cholesterol (LDL-C), triglycerides (TG), and total bile acids (TBA) [55]. During modelling, the research team employed a random forest model alongside the LASSO algorithm to screen these seven significantly valuable and mutually complementary diagnostic markers from 64 clinical features, aiming to enhance diagnostic accuracy through multidimensional information [55]. The model constructed using these complementary biomarkers, selected from extensive clinical features via random forest and LASSO algorithms, achieved an AUC of 0.947 (while 95%CI from 0.925 to 0.968) in internal validation and demonstrated robust stability in independent external validation (AUC = 0.925). t-SNE visualization further demonstrated the model’s efficacy in distinguishing cholangiocarcinoma from benign biliary tract diseases [55]. The model is now operational on an online predictive platform, offering clinicians rapid, non-invasive diagnostic support—particularly valuable when imaging or cytology results remain inconclusive [68,69]—and holds considerable potential for clinical application.

#### 4.2.2. AI-Driven Whole-Genome Repetitive Sequence Analysis and Its Application in the Diagnosis of CCA

For a long time, repetitive sequences comprising over half of the human genome were regarded as “junk DNA” or “dark matter” due to the difficulty in sequencing and analyzing them [70]. By performing whole-genome sequencing on repetitive sequences obtained through liquid biopsy, AI can uncover sequence information that is difficult to analyse using traditional methods. However, Akshaya V Annapragada’s research team developed the Analysis of RepeaT EleMents in dISease (ARTEMIS) technique. This technology employs a genome-wide approach that does not require traditional analysis workflows dependent on gene alignment. Instead, it de novo identifies billions of short sequences (kmer) within whole-genome sequencing data, revealing their enrichment in genes commonly mutated in human cancers. These repetitive sequences—including LINE, SINE, LTR, and satellite DNA—comprise over half the human genome. They undergo significant alterations during cancer progression due to genomic instability and epigenetic modifications. ARTEMIS integrates changes in the abundance of these repetitive elements through machine learning models, generating a comprehensive ARTEMIS score system that enables precise assessment of cancer development [71].

With regard to the diagnosis and application of CCA, this study provides direct data support. Utilizing a multi-cancer patient sample comprising seven cancer types (including CCA), researchers employed a model constructed by ARTEMIS in combination with another fragmentomics technique (DELFI) to conduct tissue origin analysis on all detected cancer samples. Results demonstrated that for successfully detected CCA patients (n = 24, the total number of patients with various types of cancer is 211), the model correctly traced 79% of cases to the biliary tract system (while 95% CI from 58% to 93%), achieving top-tier first-prediction accuracy among all cancer types [71]. Thus, by mining previously overlooked repetitive genomic information, the ARTEMIS technology offers a highly promising artificial intelligence analytical approach for non-invasive early diagnosis, tissue origin tracing, and future efficacy monitoring of CCA.

### 4.3. AI-Assisted Validation of In Vitro Diagnostic Results for CCA

Circulating tumor DNA mutations detected by liquid biopsy need to be validated at the histopathological level to assess their reliability as in vitro diagnostic markers. Artificial intelligence can assist in this validation process by analyzing histopathological slides. Julien Calderaro’s research team has developed a deep learning-based classification method to address the diagnostic and therapeutic challenges posed by mixed hepatocellular-cholangiocarcinoma (cHCC-CCA). The model can reclassify morphologically mixed tumors into subtypes closer to either hepatocellular carcinoma (HCC) or intrahepatic cholangiocarcinoma (iCCA) based on routine haematoxylin and eosin (HE) stained histopathological sections. Crucially, it highlights a strong association with the clinical and molecular characteristics of cholangiocarcinoma. The research employed a multi-instance learning framework incorporating attention mechanisms to construct the model. Building upon its successful differentiation between pure HCC and iCCA (AUROC > 0.99, 95% CI: 0.98–1.00), the model analyzed 405 cHCC-CCA cases, definitively classifying the majority into either HCC-predominant or iCCA-predominant categories [72]. This enhanced the assessment of patient prognosis and aided in determining whether tumors were genetically closer to hepatocellular carcinoma or cholangiocarcinoma.

The model’s predictions highly correlate with molecular alterations specific to cholangiocarcinoma. Genomic analyses reveal that the vast majority of typical iCCA-driving genetic alterations, such as *FGFR2* fusions and *IDH1/2* mutations, are enriched in tumors classified by the model as having an iCCA predisposition [73,74,75]. Spatial transcriptomics further confirmed that iCCA-like regions identified by the model indeed overexpressed cholangiocarcinoma epithelial markers (e.g., KRT7, EPCAM), functionally validating the biological basis of the classification [72].

This study confirms that deep learning can achieve molecular-guided reclassification of cHCC-CCA based on conventional pathological images. The identified iCCA-prone subtypes not only exhibit poorer prognosis but also carry targetable characteristic gene mutations for iCCA. These findings are highly consistent with the core targets of liquid biopsy ctDNA testing. Consequently, this pathological AI model can provide histological validation of ctDNA in vitro diagnostic results.

### 4.4. Synergy Between AI in CEUS Imaging and In Vitro Diagnostics

Although CEUS-based smart diagnostics do not fall within the scope of in vitro diagnostics, their combined use with liquid biopsy biomarkers is worthy of attention. Recently, numerous studies have undertaken systematic research into the intelligent diagnosis of contrast-enhanced ultrasound (CEUS), with core findings primarily manifested across two dimensions.

Firstly, concerning the construction and validation of a multi-classification diagnostic system for focal liver lesions. The team developed the multi-modal fusion model, Model-DCB, designed to achieve automatic classification of six categories of focal liver lesions, including hepatocellular carcinoma (HCC), iCCA, liver metastases, hepatic haemangioma, liver abscess, and others. This study integrated CEUS videos, selected immunohistochemical markers, and clinical information from 3725 cases across multiple centers. A two-stage strategy was employed to construct the multimodal fusion model, Model-DCB: In the first stage, separate modules for lesion classification, biomarker prediction, and clinical information screening were developed using dual-stream convolutional networks and LSTM, respectively. In the second stage, a multilayer perceptron integrated these modules and expanded the classification scope to six categories of FLL. Independent external validation demonstrated that Model-DCB achieved diagnostic accuracy (0.85–0.86) significantly superior to junior physicians (accuracy from 0.79 to 0.85), matching the proficiency of senior physicians. It also effectively enhanced the diagnostic capabilities of junior physicians, showcasing its potential in promoting diagnostic consistency at primary care levels [76].

Secondly, the development and application of a specialised diagnostic model for iCCA. Addressing iCCA—a challenging condition in differential diagnosis—the team further developed a dedicated deep learning diagnostic model. Through retrospective analysis of 1148 CEUS cases from multiple centers and employing algorithms such as ResNet-50, the model demonstrated excellent external validation performance (AUC = 0.92). A multicenter comparative study confirmed that the diagnostic performance of this iCCA-specific model (AUC = 0.91) was comparable to that of senior CEUS specialists (AUC = 0.87) and senior MRI specialists (AUC = 0.89), while significantly outperforming junior and intermediate-level practitioners. Similarly, the model effectively assisted junior and intermediate physicians, elevating their diagnostic AUCs from 0.72 to 0.89 and from 0.78 to 0.90, respectively, achieving expert-level performance [77].

Although CEUS-AI is not strictly a diagnostic tool, it can serve as a complementary tool to liquid biopsy; for example, when liquid biopsy results are inconclusive from an imaging perspective, CEUS-AI can provide additional imaging evidence. In the future, the multimodal fusion of imaging omics features with liquid biopsy data (such as ctDNA and EVs) is expected to further improve diagnostic accuracy.

It must be emphasized, however, that current CEUS-based AI models have been developed and validated exclusively for intrahepatic lesions, and their applicability to eCCA—which present distinct sonographic challenges—remains unexplored. Consequently, the synergy between CEUS-AI and liquid biopsy described here pertains specifically to iCCA, and separate imaging-AI strategies will be required for the extrahepatic subtypes.

### 4.5. The Application of AI in Uncovering the Tumor Microenvironment of CCA

In analyzing the highly heterogeneous tumor microenvironment of cholangiocarcinoma, AI is advancing precision medicine through two complementary approaches: multi-omics in situ mapping and imaging radiomics decoding.

In the field of spatial in situ mapping, Xuanwen Bao’s research team has integrated imaging mass spectrometry cytology, spatial proteomics, spatial transcriptomics, and single-cell RNA sequencing. They have, for the first time, systematically mapped the spatial tumor microenvironment (TME) of iCCA at single-cell resolution. This work has identified spatially organized structures closely linked to prognosis, such as the contiguous distribution of CD163^+^ M2-like macrophages and CD8^+^ T cells. Building upon this, the team further developed a deep learning-based prognostic prediction model capable of highly accurate survival forecasting using merely 1 mm^2^ of tumor tissue samples (the mean accuracy reached 98.57%), highlighting AI’s capacity to extract insights from spatial microenvironmental information [78].

Another category of AI methods based on conventional medical imaging also provides a significant complement to decoding the TME. Gu-Wei Ji’s research team developed an AI model that first utilized techniques such as spatial transcriptomics to identify an immune score comprising three key genes, including PLAUR. Subsequently, from thousands of radiomics features, three manually designed radiomics features were ultimately selected through algorithms such as recursive feature elimination. A final predictive model was constructed using logistic regression [79]. The study revealed that these features (e.g., wavelet-LHL_gldm_MCC) quantify tumor intra-lesional heterogeneity, reflecting tumor biological behavior at different scales [80]. The clinical utility of this model is substantial. In terms of prognosis, its risk score serves as an independent predictor of survival duration (C-index > 0.64), outperforming conventional indicators [79]. Regarding treatment response prediction, its accuracy in forecasting responses to immune checkpoint inhibitor plus chemotherapy (AUC = 0.84, 95% CI 0.69–0.99) significantly surpasses PD-L1 testing [81]. More importantly, for patients predicted to have poor response, studies indicate that targeting uPAR enhances anti-PD-1 efficacy, offering novel strategies to overcome resistance [82]. This research confirms AI’s capability to decode tumor microenvironment information from routine CT imaging, demonstrating significant translational value in iCCA prognosis assessment, treatment response prediction, and combined therapy exploration. It signifies AI’s emergence as a vital component within the precision medicine framework for CCA.

### 4.6. Comprehensive Application of AI in Precision Diagnosis and Treatment of CCA

In terms of diagnosis, this paper elaborates on the specific applications of artificial intelligence in the imaging and pathological analysis of cholangiocarcinoma. By employing deep learning algorithms to extract features from CT, MRI, and contrast-enhanced ultrasound images, AI models can identify minute lesions that are often missed by traditional methods. They can precisely measure tumor size, morphology, and the tumor’s relationship with surrounding tissues, thereby aiding physicians in achieving a more accurate and earlier diagnosis of CCA.

In terms of molecular stratification, this paper demonstrates how artificial intelligence integrates multi-omics data (genomics, transcriptomics, proteomics, etc.) and radiomic features to identify molecular subtypes of cholangiocarcinoma. By utilizing machine learning algorithms to uncover correlations between routine pathological images or radiomic features and driver gene alterations, AI can classify patients into subtypes associated with distinct prognoses and treatment responses.

In terms of treatment monitoring, this paper describes how artificial intelligence, by analyzing the dynamic changes in liquid biopsy biomarkers, enables real-time assessment of treatment response and recurrence risk. AI models analyze the concentration and evolving characteristics of ctDNA, CTCs, and EVs to predict disease recurrence earlier than conventional imaging and dynamically track tumor clonal evolution and the emergence of drug-resistant mutations. Concurrently, multimodal fusion models integrate multi-omics information from liquid biopsies to establish a closed-loop “data acquisition-intelligent analysis” system, offering a powerful tool for clinicians to promptly adjust therapeutic strategies and avert disease progression.

### 4.7. Liquid Biopsy and AI Integration

**Figure 2:**
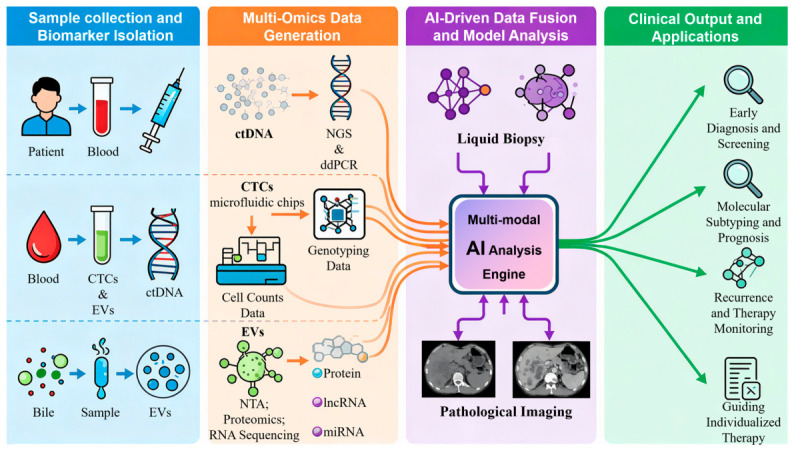
Precision Therapy for Future Cholangiocarcinoma—Workflow Diagram Integrating Liquid Biopsy Multi-Omics Data and AI.

This study overcomes the limitations of existing reviews, which typically discuss liquid biopsy and AI technology in cholangiocarcinoma separately, by innovatively and systematically integrating their synergistic application. A schematic overview of this integrated precision medicine framework is provided in Figure 2**.** The core of this fusion lies in leveraging AI’s exceptional computational and pattern recognition capabilities to thoroughly analyze the high-dimensional, heterogeneous information generated by liquid biopsy. Specifically, AI constructs multimodal large models to integrate multiple datasets—including ctDNA mutation profiles, nucleic acids and proteins carried by EVs, and CTCs counts and phenotypes—thereby uncovering biomarker combinations unattainable through single markers [57,83]. This enables comprehensive identification of tumor heterogeneity, with its methodological foundations established by cross-organ recognition models such as CHIEF [84].

Building upon this foundation, AI can directly enhance the detection efficacy of liquid biopsies by learning deep features within the data. For instance, by analyzing the fragmentomics characteristics of cfDNA, AI can significantly improve sensitivity in detecting ctDNA signals at low abundance levels. This approach aligns with the methodology employed by ARTEMIS technology in uncovering genomic “dark matter” [71]. Furthermore, AI models can dynamically track biomarker evolution pathways, potentially indicating tumor recurrence earlier than imaging modalities [49]. Although challenges remain in data standardization and generalization capabilities, the synergistic advancement of these technologies will undoubtedly propel cholangiocarcinoma diagnosis and treatment towards a new era characterized by intelligence, dynamism, early intervention, and precision.

### 4.8. Limitations of AI in the In Vitro Diagnosis of CCA

Although AI has demonstrated significant potential in the in vitro diagnosis of CCA, its translation from the laboratory to clinical practice still faces multifaceted challenges.

Firstly, AI-driven in vitro diagnostics face stringent regulatory approval requirements: in 2025, the US FDA issued new guidance on AI devices, requiring a full lifecycle review of algorithm development, validation and updates [85]; the EU AI Act classifies most medical AI as high-risk systems, mandating six mandatory compliance frameworks—including risk management, data governance and transparency—and requiring mandatory compliance certification. Complex cross-regional regulations significantly increase the difficulty and time costs associated with market entry. Integrating the AI Act with the current In Vitro Diagnostic Regulation (IVDR) still holds promise for promoting innovation whilst enhancing patient safety. Regulatory bodies can reduce duplication of effort and improve efficiency by harmonizing standards and streamlining compliance reviews. To establish a regulatory framework that balances innovation and safety, it is essential to foster collaborative participation from multiple stakeholders, including industry, the medical community and patient organizations [86,87].

Secondly, the development, validation and deployment of AI models are costly: a review indicates that only 28% of health economics studies take implementation costs into account, and most AI systems remain in the early stages of development [88]. The investment required across the entire chain—from data annotation and computing resources to subsequent model maintenance and updates—is substantial. For rare cancers such as CCA, it is difficult to spread the costs through large-scale commercialization, which further exacerbates the challenges of widespread adoption. To address this, a multi-party cost-sharing mechanism should be established, and research and development costs across the entire chain should be reduced by optimizing algorithm efficiency and leveraging open-source tools.

Furthermore, the reproducibility crisis has long been a major challenge in AI medical research: deep neural networks are extremely sensitive to even minor variations in data and hardware, and even sharing code does not guarantee that results will be entirely consistent [89]; research has found that fewer than 4% of machine learning studies use data from different clinical settings for external validation, making it difficult to assess the reliability of most models [90]. To this end, efforts should be made to establish a standardized algorithm evaluation framework and a mandatory multicenter external validation mechanism to enhance the reproducibility and reliability of model results.

The lack of sufficient samples and data hinders the standardization of AI models. Given the low incidence of cholangiocarcinoma, the scale of data available for training AI models is extremely limited. Training on small datasets can easily lead to overfitting, whereby models perform well in internal validation but often show a significant decline in performance on external, independent datasets; furthermore, the lack of data standardization across different institutions further limits the models’ generalization ability [91]. To address this, a multicenter data-sharing collaboration network should be established, and efforts should be made to promote standardized protocols for data collection and processing, while simultaneously developing robust modeling strategies suitable for small samples.

In terms of clinical translation, there is a dual gap between research and clinical application in the areas of compliance and regulation. Some standard tools that meet medical device standards are implemented without seeking regulatory authorization, whilst other models that influence clinical decision-making are deployed without any external approval [92]. Furthermore, for diseases such as CCA, where sample sizes are limited, conducting prospective clinical validation studies is particularly challenging. Therefore, it is necessary to establish a tiered and categorized regulatory framework, create a dedicated fast-track process for clinical validation and approval of treatments for CCA, and strengthen compliance guidance for the clinical translation phase.

Integrating AI into in vitro diagnostics requires multidisciplinary expertise spanning molecular biology, bioinformatics and data science. Currently, medical schools generally lack relevant training programmes for doctors, and most doctors do not possess the technical skills required to manage AI-driven diagnostic workflows. The absence of a common language within multidisciplinary teams, coupled with communication barriers between clinicians and data scientists, directly impedes the effective alignment of AI models with clinical needs. Thus, the medical education curriculum should be reformed to include interdisciplinary training in AI and data science, and a regular communication mechanism should be established to foster collaboration between clinicians and data scientists.

At the same time, equipment and infrastructure are practical considerations that must be taken into account. The deployment of AI systems requires supporting computing resources, data storage and processing capabilities, which are often severely lacking in healthcare institutions with limited technical infrastructure. Furthermore, the ‘black box’ nature of AI models makes it difficult for clinicians to understand and trust their diagnostic recommendations [93]. To tackle this, it is necessary to strengthen the IT infrastructure of primary care facilities and develop explainable AI technologies to enhance the transparency and clinical acceptability of model-based decisions.

Finally, the practical application of AI diagnostic tools is constrained by the policy environment. The rapid evolution of medical AI technology has far outpaced the ability of existing policies and regulations to keep pace. Differences and inconsistencies in regulations across regions make it difficult to roll out AI-powered in vitro diagnostic devices rapidly across multiple markets. The lag in medical insurance reimbursement policies also limits the clinical adoption of AI diagnostic tools—even when the technology has been approved, the lack of a clear reimbursement pathway makes it difficult for it to become part of routine clinical practice [94]. Accordingly, it is necessary to promote the dynamic updating and cross-regional coordination of policies and regulations, and to establish a mechanism for evaluating and incorporating AI diagnostic technologies into health insurance coverage that keeps pace with their development.

## 5. Discussion

The high mortality rate of CCA is linked to the difficulty in diagnosing the disease. Traditional diagnostic methods often make it difficult to confirm the diagnosis due to their low sensitivity and the risks associated with invasive procedures. This review outlines the transformative impact of liquid biopsy and AI on early diagnosis. Liquid biopsy provides a robust means for non-invasive, dynamic, and comprehensive profiling of tumor molecular markers by detecting biomarkers such as CTCs, EVs, ctDNA, and CLU in peripheral blood. This technology not only holds promise for providing early warning of CCA but also shows potential for evaluating treatment efficacy, detecting micrometastases, and monitoring for recurrence. However, the clinical application of liquid biopsy in CCA still faces critical challenges. These primarily include high costs, limited insurance coverage, and difficulties in adoption at primary care settings; insufficient sensitivity for tumors with low shedding rates; a lack of standardized protocols and quality control systems, leading to poor inter-platform consistency; and the limited clinical utility of positive results, which are not yet sufficient to guide treatment decisions independently and must be integrated with conventional methods [54,95]. Moreover, the anatomical and molecular heterogeneity between iCCA and eCCA necessitates a more nuanced interpretation of liquid biopsy results. Future clinical guidelines and validation studies must account for this subtype specificity to avoid overgeneralization of diagnostic performance.

Meanwhile, AI, by deeply mining information from pathological images and genomic data, has enhanced the objectivity, efficiency, and accuracy of diagnosis and has even enabled, to some extent, the prediction of key gene mutations. At the same time, we need to be aware that: AI diagnosis of CCA is limited by data quality, lack of interpretability, and single-task focus; it currently serves only as an auxiliary tool and cannot replace comprehensive clinical decision-making [96].

Although liquid biopsy continues to face challenges related to standardization, sensitivity, and clinical validation, its deep integration with AI has shown substantial promise in shaping a future precision medicine framework for CCA. Future efforts should focus on fostering multi-disciplinary collaboration, establishing unified standard operating procedures, and advancing the application of this approach from late-stage monitoring to the early screening of high-risk populations. Such advances will provide a practical pathway toward achieving accurate diagnosis, timely intervention, and personalized treatment strategies for CCA.

## Figures and Tables

**Table 1:** Comparison of Specific Diagnostic Values for CCA Using Traditional Methods.

Radiological Imaging or Biomarker	AUC	Sensitivity	Specificity	Note	Reference
US	0.93 (95% CI: 0.87–0.99)	88% (95% CI: 84–90%)	80% (95% CI: 78–83%)	Achieving complete visualization of the extrahepatic bile ducts is challenging.	[18]
CT	0.85 (95% CI: 0.82–0.88)	87% (95% CI: 81–91%)	82% (95% CI: 77–86%)	It has limitations in delineating biliary anatomy in complex hilar lesions.	[19]
MRI	0.93 (95% CI: 0.90–0.96)	81% (95% CI: 79–84%)	90% (95% CI: 88–91%)	Is contraindicated in patients with certain metallic implants.	[18]
CA19-9	0.83	62%	63%	There is some heterogeneity among studies, and the diagnostic performance of CA19-9 for cholangiocarcinoma is influenced by multiple factors. For example, false positives may occur in benign biliary diseases (cholangitis, biliary obstruction), while false negatives may arise in Lewis antigen-negative populations.	[20]
CEA	0.541	9.0%	99.2%	When CEA is used alone, its diagnostic value for CCA is limited. However, when combined with CA125 and CA19-9, the diagnostic performance is significantly enhanced.	[21]
CEA + CA125 + CA19-9	0.888	85.1%	83.1%		[22]

**Table 2:** Diagnostic Performance Comparison of Bile EVs with Other Biomarkers.

Biomarker	Sample Source	ROC-AUC	Sensitivity	Specificity	Accuracy
Biliary EVs concentration	Bile	1.000	100%	100%	100%
Serum CA19-9	Serum	0.711	66.7%	80.0%	73.3%
Biliary CEA M6 protein	Bile	0.964	100%	80.0%	90.0%
Serum EVs concentration	Serum	0.813	46.7%	80.0%	63.3%

Note: ROC-AUC: Accuracy in distinguishing malignant from benign stenosis.

**Table 3:** Primary Functions of EVs-Derived Protein Markers in Cholangiocarcinoma.

Biomaker Panel	Clinical Application	AUC	Primary Function	Reference
CRP + FRIL	Pan-CCA Diagnosis	0.941	Unaffected by the cause of the disease, enabling early diagnosis; readily detectable in total serum.	[40]
CRP + FIBRINOGEN + FRIL	Diagnosis of CCA associated with PSC	0.947	Specifically designed to identify patients with Primary Sclerosing Cholangitis (PSC) who have developed cancer, particularly suitable for early detection.	
CRP + PIGR + VWF	The diagnosis of CCA not related to PSC	0.992	Can almost perfectly distinguish disseminated CCA from healthy individuals	
CRP + FGL1	Differentiating iCCA from HCC	0.959	Effectively addresses clinical differential diagnosis challenges, demonstrating significant superiority over existing approaches.	
FIBG/FGL1/PIGR/VWF/OIT3	Auxiliary diagnosis	A single AUC lies between 0.6 and 0.8.	As a key individual biomarker, it forms the basis of the aforementioned combination; enriched in both EVs and whole serum.	

**Table 4:** Comparative Analysis of Non-coding RNAs in Cholangiocarcinoma-Associated Exosomes.

Marker	Test Sample	Primary Mechanism of Action	Clinical Application Potential	References
TTN-AS1 (lncRNA)	serum	Following uptake by CCA cells, it promotes cell cycle progression by upregulating Cyclin D1 and induces epithelial–mesenchymal transition, thereby enhancing cellular proliferation, migration and metastatic potential.	Serum exosomes exhibited the highest AUC (0.889), demonstrating significant correlation with lymph node metastasis and TNM staging, thereby serving as a favorable diagnostic biomarker.	[42]
LINC01812 (lncRNA)	Tissue and cell culture supernatants	Upon phagocytosis by macrophages, it binds to the TUBB4B protein, activates the Notch signalling pathway, induces M2 macrophage polarization, and secretes CCL2, thereby promoting tumor neuroinvasion.	Its expression is significantly correlated with nerve invasion, serving as a potential indicator for predicting PNI and adverse outcomes, and may function as a favorable prognostic biomarker.	[43]
miR-182-5p/miR-183-5p (miRNA)	bile	Following uptake by CCA cells, targeted inhibition of HPGD gene expression reduces prostaglandin E2 degradation, leading to PGE2 accumulation within the tumor microenvironment. This enhances tumor stem cell properties and drives tumor progression.	As a non-invasive bile liquid biopsy target, it holds potential for diagnosing and evaluating tumor stem cell characteristics, serving as a promising diagnostic biomarker.	[44]

**Table 5:** Overview of liquid biopsy biomarkers for cholangiocarcinoma.

Biomarker	Sample Source	Main Target/Analyte	Core Technology	Primary Application in CCA	Current Limitations
CTCs	Peripheral blood	EpCAM^+^ tumor cells	CellSearch system; microdroplet digital platform	Prognostic assessment; metastasis risk evaluation	Low abundance in early-stage disease; sensitivity depends on enrichment methods; most validation studies have not stratified results by iCCA versus eCCA subtypes.
EVs	Serum, bile	Proteins, miRNAs, lncRNAs, circRNAs	Ultracentrifugation, TEM, NTA, Western blot	Early diagnosis (especially bile EVs); differential diagnosis	Lack of standardized isolation and storage protocols; diagnostic performance of bile EVs is well established mainly for eCCA, whereas optimal EVs sources for iCCA require further definition.
ctDNA	Plasma	Tumor-specific mutations (*FGFR2*, *IDH1*, *KRAS*, *TP53*)	ddPCR; NGS	MRD detection; recurrence surveillance (earlier than imaging)	Low sensitivity in early-stage tumors (clinical sensitivity < 50%); detection rates vary by subtype depending on the prevalence of targetable mutations.
CLU	Bile	Clusterin protein	Western blot; SPR-based fiber optic biosensor	Real-time in vivo diagnosis combined with endoscopy	Requires endoscopic access; limited clinical validation in small single-center cohorts; multicenter studies needed; bile-based testing is inherently more applicable to extrahepatic disease.

## Data Availability

No new data were created or analyzed in this study. Data sharing is not applicable to this article.

## Abbreviations

The following abbreviations are used in this manuscript:

CCA	Cholangiocarcinoma
CTCs	Circulating tumor cells
EVs	Extracellular vesicles
ctDNA	Circulating tumor DNA
CLU	Clusterin
AI	Artificial Intelligence
iCCA	Intrahepatic cholangiocarcinoma
pCCA	Perihilar cholangiocarcinoma
dCCA	Distal cholangiocarcinoma
eCCA	Extrahepatic cholangiocarcinoma
US	Ultrasound
CT	Computed tomography
MRI	Magnetic resonance imaging
CEA	Carcinoembryonic antigen
MRD	Molecular residual disease
ddPCR	Droplet digital PCR
NGS	Next-generation sequencing
PSC	Primary sclerosing cholangitis
HCC	Hepatocellular carcinoma
CEUS	Contrast-enhanced ultrasound
ARTEMIS	Analysis of RepeaT EleMents in dISease
TME	Tumor microenvironment
